# Clinical and Imaging Characteristics to Discriminate Between Complicated and Uncomplicated Acute Cholecystitis: A Regression Model and Decision Tree Analysis

**DOI:** 10.3390/diagnostics15141777

**Published:** 2025-07-14

**Authors:** Yu Chen, Ning Kuo, Hui-An Lin, Chun-Chieh Chao, Suhwon Lee, Cheng-Han Tsai, Sheng-Feng Lin, Sen-Kuang Hou

**Affiliations:** 1 Department of Emergency Medicine, Taipei Medical University Hospital, Taipei 110, Taiwan; b101102093@tmu.edu.tw (Y.C.); sevenoking219@gmail.com (H.-A.L.); chaosees@gmail.com (C.-C.C.);; 2 Department of Emergency Medicine, School of Medicine, College of Medicine, Taipei Medical University, Taipei 110, Taiwan; 3 Department of Public Health, School of Medicine, College of Medicine, Taipei Medical University, Taipei 110, Taiwan; 4 School of Public Health, College of Public Health, Taipei Medical University, Taipei 110, Taiwan; 5 Center of Evidence-Based Medicine, Taipei Medical University Hospital, Taipei 110, Taiwan; 6 Department of Medical Research, Taipei Medical University Hospital, Taipei 110, Taiwan; 7 TMU Research Center for Digestive Medicine, Taipei Medical University, Taipei 110, Taiwan

**Keywords:** complicated cholecystitis, Hounsfield unit, gallbladder wall thickness, gallbladder gangrenous change, gallbladder volume

## Abstract

**Background**: Acute complicated cholecystitis (ACC) is associated with prolonged hospitalization, increased morbidity, and higher mortality. However, objective imaging-based criteria to guide early clinical decision-making remain limited. This study aimed to develop a predictive scoring system integrating clinical characteristics, laboratory biomarkers, and computed tomography (CT) findings to facilitate the early identification of ACC in the emergency department (ED). **Methods**: We conducted a retrospective study at an urban tertiary care center in Taiwan, screening 729 patients who presented to the ED with suspected cholecystitis between 1 January 2018 and 31 December 2020. Eligible patients included adults (≥18 years) with a confirmed diagnosis of acute cholecystitis based on the Tokyo Guidelines 2018 (TG18) and who were subsequently admitted for further management. Exclusion criteria included (a) the absence of contrast-enhanced CT imaging, (b) no hospital admission, (c) alternative final diagnosis, and (d) incomplete clinical data. A total of 390 patients met the inclusion criteria. Demographic data, laboratory results, and CT imaging features were analyzed. Logistic regression and decision tree analyses were used to construct predictive models. **Results**: Among the 390 included patients, 170 had mild, 170 had moderate, and 50 had severe cholecystitis. Key predictors of ACC included gangrenous changes, gallbladder wall attenuation > 80 Hounsfield units, CRP > 3 mg/dL, and WBC > 11,000/μL. A novel scoring system incorporating these variables demonstrated good diagnostic performance, with an area under the curve (AUC) of 0.775 and an optimal cutoff score of ≥2 points. Decision tree analysis similarly identified these four predictors as critical determinants in stratifying disease severity. **Conclusions**: This CT- and biomarker-based scoring system, alongside a decision tree model, provides a practical and robust tool for the early identification of complicated cholecystitis in the ED. Its implementation may enhance diagnostic accuracy and support timely clinical intervention.

## 1. Introduction

Acute cholecystitis, an inflammation of the gallbladder (GB), is a common disease among patients presenting to the emergency department (ED) with abdominal pain that requires hospitalization and surgery [1]. Without timely treatment, patients with acute cholecystitis may develop complications such as gangrene, perforation, or emphysematous change. Acute complicated cholecystitis (ACC) is associated with a long average length of stay (LOS) and high morbidity and mortality [2,3,4].

In the ED, ultrasound and abdominal computed tomography (CT) are the two major tools used to diagnose cholecystitis. Ultrasound is used in the ED for the rapid screening of cholecystitis, with sensitivity ranging within 68–92% and specificity ranging within 80–88%. However, the diagnostic performance by ultrasound is operator-dependent and, in the case of ACC, the sensitivity decreases to 50–70% because of the poor detection of pericholecystic changes [5,6,7]. Mika et al. [8] highlighted the low sensitivity of ultrasound in detecting ACC, further emphasizing the need for alternative or adjunctive diagnostic strategies in this setting. This diagnostic limitation underscores the clinical gap addressed in this study and supports the need for more objective and reliable methods to assess severity.

CT is the reference standard for discriminating between complicated and uncomplicated acute cholecystitis [6]. CT images of ACC indicate intraluminal findings (sloughed mucosa, hemorrhage, and abnormal gas), GB wall abnormalities (fat-stranding, asymmetric wall thickening, abnormal gas, and contrast enhancement), and pericholecystic changes (gangrenous necrosis, emphysematous change, pericholecystic fluid, abscess formation, and GB perforation) [2,9]. Recent studies have revealed GB distention and GB wall thickening, and high CT density values of the GB wall in CT images were associated with a high risk of ACC [9,10,11,12,13,14]. Traditionally, CT image features such as a “distended gallbladder” or “thickened wall” are described using subjective, qualitative terms. In this study, we converted these subjective descriptors into measurable, numerical values (e.g., a specific wall thickness in millimeters, Hounsfield units for density). This digitization improves objectivity, facilitates standardized interpretation, and enhances communication between emergency physicians, radiologists, and surgeons, thereby supporting the development of a practical scoring system to predict complicated cholecystitis.

In general, several treatment methods, including antibiotics, percutaneous transhepatic gallbladder drainage (PTGBD), and cholecystectomy are used for acute cholecystitis. In patients with moderate to severe cholecystitis, PTGBD with delayed cholecystectomy is associated with a lower rate of open cholecystectomy, less intraoperative bleeding, a shorter duration of postoperative abdominal drainage, shorter hospital stays after cholecystectomy, and a lower incidence of respiratory failure [15,16,17,18]. In the Tokyo Guidelines 2018 (TG18), the severity assessment of acute cholecystitis contains various parameters, including most laboratory biomarkers, along with some radiographic features. However, we still lack a convenient tool to precisely evaluate the image characteristics, which makes the severity assessment and treatment strategies for acute cholecystitis primarily dependent on the personal experience of the surgeons.

There is limited research proposing how imaging findings can be objectively evaluated to aid surgeons in their decision-making. In this study, we investigated the general characteristics, laboratory biomarkers, and CT findings of those presenting to the ED with a diagnosis of acute cholecystitis, aiming to develop a new scoring system to predict ACC at an early stage.

## 2. Methods

### 2.1. Definition of Acute Complicated Cholecystitis (ACC)

In this study, we defined acute complicated cholecystitis (ACC) based on the Tokyo Guidelines 2018 (TG18) severity grading system. Patients with moderate (Grade II) or severe (Grade III) acute cholecystitis were classified as having ACC. Grade II is characterized by clinical findings such as leukocytosis (>18,000/mm^3^), palpable right upper quadrant mass, symptom duration of >72 h, or marked local inflammation (e.g., gangrene, abscess, or peritonitis). Grade III includes systemic organ dysfunction, such as cardiovascular, neurological, respiratory, renal, hepatic, or hematological impairment. Patients not meeting the criteria for Grade II or III were classified as having mild (Grade I) acute cholecystitis and were considered as the uncomplicated group.

### 2.2. Study Design and Data Collection

This single-center, retrospective study was conducted using the Health Information System of Taipei Medical University Hospital (TMUH) between 1 January 2018 and 31 December 2020, at the emergency department (ED) of TMUH, a tertiary care center in Taipei, Taiwan. The ED is staffed by board-certified emergency physicians and handles approximately 60,000 patient visits annually. Patients were treated according to standard clinical practices. Eligible participants were identified using the International Classification of Diseases, Tenth Revision, Clinical Modification (ICD-10-CM) codes (Table S1). Adults aged 18 years or older who presented to the ED with a confirmed diagnosis of acute cholecystitis, as defined by the Tokyo Guidelines 2018 (TG18) diagnostic criteria, and who were subsequently admitted to a hospital ward were included. Cases included patients with or without gallstones, and some may have been complicated by concurrent cholangitis or pancreatitis.

Patients were excluded if they had no CT imaging or only a non-contrast-enhanced CT scan, were not admitted to the ward (including those discharged directly from the ED or against medical advice), lacked a final diagnosis of acute cholecystitis, or had missing clinical data. Data for eligible patients were accessed for research purposes between 1 April 2023 and 30 June 2023. Enrolled patients were stratified into mild, moderate, and severe groups based on the TG18 severity grading system (Table S2). This study was approved by the Institutional Review Board of Taipei Medical University (Ref. No. N202303059) on 28 March 2023. As a retrospective analysis of anonymized, registry-based data, the requirement for informed consent was waived.

### 2.3. Variables and Biomarkers

The data on the patients’ age, sex, vital signs, and Glasgow coma scale score (GCS) on arrival at the ED were recorded. Other data included laboratory testing data at the ED, history of medical comorbidities, and data on the treatments and outcomes after admission. We also analyzed the Charlson comorbidity index (CCI) and the grades of acute cholecystitis among the patients according to TG18.

### 2.4. CT Imaging Protocol

Abdominal multidetector computed tomography (MDCT) imaging was performed using a 128-slice SOMATOM Perspective scanner (Siemens Healthineers Inc., Forchheim, Germany). Scanning ranged from the top of the liver to the symphysis pubis using a 0.625 mm spiral section in the portal venous phase, approximately 70–80 s after the intravenous injection of 95 mL of Optiray 350 contrast medium (Mallinckrodt Medical Inc., Pointe Claire, QC, Canada). This phase provides optimal enhancement of the solid organs, bowel, and vasculature. Initial CT interpretations were conducted by on-duty emergency physicians. All original images were later reviewed by four emergency department physicians (Y.C., N.K., S.L., C.-H.T.) and the findings were cross-validated with the formal report by the board-certified abdominal radiologists to ensure accuracy and consistency.

### 2.5. CT Features Related to Acute Cholecystitis

**Figure 1:**
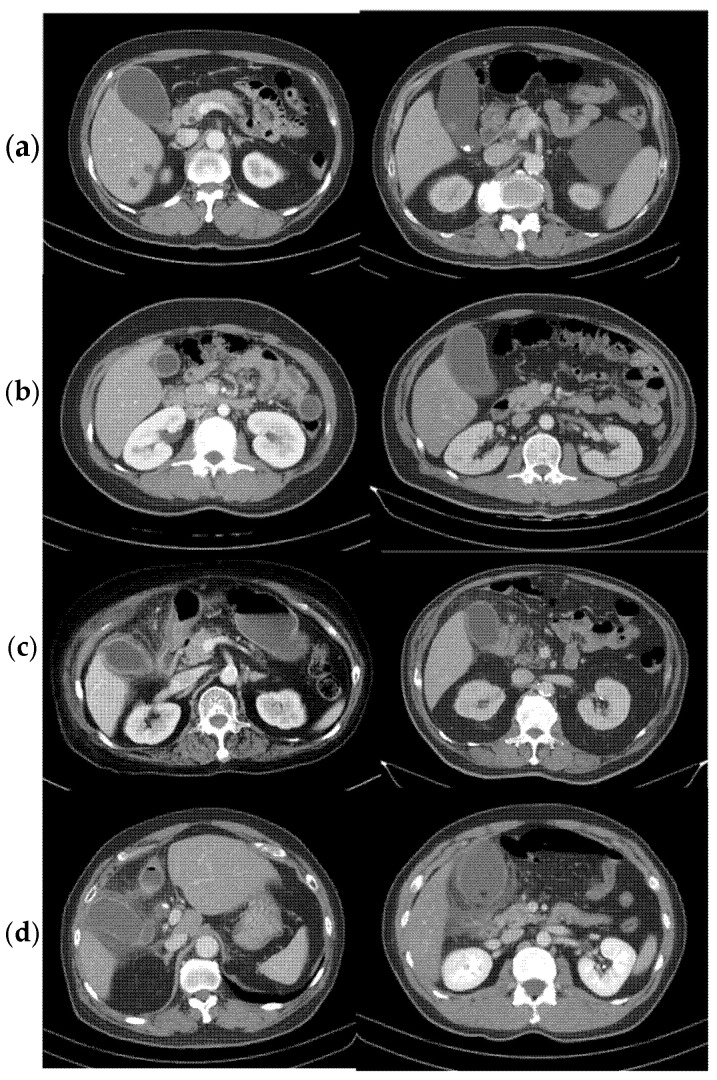
Image example. (**a**) Normal thickness of GB wall (≤3 mm), cholelithiasis of hyperattenuation of the GB wall; (**b**) thickening of the GB wall (>3 mm) with no pericholecystic fat stranding; (**c**) thin linear or ground glass pattern of pericholecystic fat stranding; (**d**) reticular pattern of pericholecystic fat stranding, abscess formation, GB perforation, and GB gangrenous change.

The CT parameters analyzed included the presence of gallstones, biliary sludge, gallbladder (GB) wall thickness, GB wall attenuation (in Hounsfield units), GB volume, pericholecystic fluid, pericholecystic fat stranding, abscess formation, GB perforation, and gangrenous changes. Definitions and criteria for these findings were established by four investigators through consensus, guided by radiologist reports and referenced imaging criteria. Gallstones, typically hyperattenuating, and biliary sludge, typically hypoattenuating, were identified on non-contrast images. GB wall thickness and the highest HU values were measured using the picture archiving and communication system (PACS) with reproducibility. GB volume was calculated as 0.52 × length (cm) × width (cm) × height (cm) [19,20], using coronal views to determine the longest (length) and widest (width) diameters, while height was derived by multiplying the number of CT slices (5 mm thickness) by 0.5 cm. Gangrenous change was characterized by low-attenuation areas, gas formation, and peri-fluid accumulation [21]. Figure 1 shows the representation of different imaging characteristics of GB: normal thickness of the GB wall (≤3 mm), only with cholelithiasis or hyperattenuation of the GB wall (Figure 1a); thickening of the GB wall (>3 mm) with no pericholecystic fluid nor fat stranding (Figure 1b); a thin linear or ground glass pattern of pericholecystic fluid and fat stranding (Figure 1c); and a reticular pattern of pericholecystic fat stranding, abscess formation, and GB perforation, with gangrenous changes on the CT (Figure 1d) [3].

### 2.6. Statistical Analysis

Analysis of variance (ANOVA) and Pearson’s chi-square test (or Fisher’s exact test) were performed to examine differences in continuous and categorical variables among the mild, moderate, and severe groups of TG18. Accordingly, we defined the mild group as the uncomplicated group, while the moderate and the severe groups were defined as the complicated group to proceed with further analysis.

Univariate binary logistic regression was used to determine the odds ratios (ORs) and the corresponding 95% confidence intervals (CIs) for the prediction of the complicated group. In the multivariable binary logistic regression analysis, we set the dependent variables as complicated versus uncomplicated groups and employed two types of models: (1) in Model 1, we used independent variables of radiographic features that showed statistical significance in univariate logistic regression, and (2) in Model 2, we used independent variables of radiographic features, in combination with general characteristics of laboratory tests that showed statistical significance in univariate logistic regression. We built a scorning system (Model 3) based on the OR from Model 2.

The predictive performance of each model was obtained by calculating the areas under the curves (AUCs) of the receiver of operating characteristic (ROC) curves. The Youden index was used to determine the optimal cutoff values. A *p*-value < 0.05 was considered statistically significant, and all statistical analyses were performed using SPSS version 26 (IBM Corp., Armonk, NY, USA).

The odds ratio in the logistic regression model only revealed the importance of each variable. To enhance its clinical applicability, we employed decision tree analysis to provide recommendations for the sequence of significant variables.

To build the decision tree for the classification of the severity of cholecystitis, we used the algorithm of classification and regression tree (CART)—a predictive model that applies a dichotomous decision process and splits data according to a certain cut-off value—which was used to establish clinical diagram to verify Model 3. In the CART model, all significant variables in the Model 3 were included. We performed the decision tree analysis using R software 4.1.19 with the rpart package (R Core Team, 2021).

## 3. Results

### 3.1. Population Characteristics

**Figure 2:**
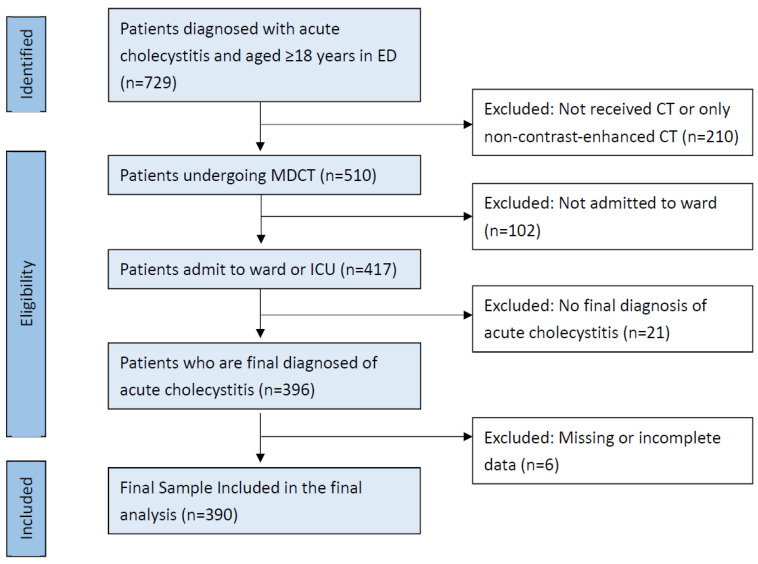
Flow chart illustrating the inclusion and exclusion criteria of the retrospective study.

A total of 729 patients were initially identified using ICD-10-CM codes (Table S1) as adults aged ≥18 years who presented to the emergency department (ED) with confirmed acute cholecystitis between 1 January 2018 and 31 December 2020. Of these, 339 patients were excluded for the following reasons: no CT scan was performed or only a non-contrast-enhanced CT was obtained (n = 210); they were not admitted to the ward, including those discharged directly from the ED or against medical advice (n = 102); they received no final diagnosis of cholecystitis (n = 21); or they had missing data (n = 6). Consequently, 390 patients were included in the final analysis (Figure 2). According to the Tokyo Guidelines 2018 (TG18) severity grading system, 170 patients were classified as having mild acute cholecystitis, 170 as having moderate cholecystitis, and 50 as having severe cholecystitis. Detailed demographic and clinical characteristics are presented in Table 1.

Significant differences were observed among the three groups in terms of mean age (*p* < 0.001), sex (*p* = 0.026), diabetes mellitus (DM; *p* < 0.001), hypertension (*p* = 0.001), and chronic kidney disease (CKD; *p* < 0.001). On physical examination, the moderate and severe groups exhibited significantly higher body temperature, heart rate, and altered Glasgow coma scale (GCS) scores (all *p* < 0.001). Additionally, the requirement for oxygen therapy, FiO2, and inotropic support during the ED stay was significantly greater in the moderate and severe groups (both *p* < 0.001).

### 3.2. Clinical Outcomes

Patients classified as severe had significantly longer hospital stays (12.54 ± 10.16 days) compared to those with moderate (7.02 ± 4.22 days) and mild (5.59 ± 2.86 days) grades of cholecystitis (*p* < 0.001). ICU admission rates rose sharply with severity, from 2.9% in the mild group to 11.8% in the moderate group and 36% in the severe group (*p* < 0.001). The need for surgical intervention was most common in the mild group (55.3%) and declined with increasing severity, reflecting a potential shift in treatment strategy or operability constraints in more critical patients (*p* < 0.001). PTGBD was more frequently performed in the moderate group (32.4%), while ERCP usage did not significantly differ among the groups (*p* = 0.52). These findings underscore the progressive burden of severe disease on resource utilization and patient outcomes.

### 3.3. Laboratory Markers and Radiographic Features

The laboratory data and parameters measured on contrast CT are presented in Table 2. The white blood cell (WBC) count (*p* < 0.001), neutrophil percentage (*p* < 0.001), C-reactive protein level (CRP; *p* < 0.001), total bilirubin level (bilirubin T; *p* = 0.013), creatinine level (*p* < 0.001), neutrophil-to-lymphocyte ratio (NLR; *p* < 0.001), and platelet-to-lymphocyte ratio (PLR; *p* < 0.001) were significantly higher in the moderate and severe groups (*p* < 0.001). The platelet count (*p* < 0.001) and lymphocyte-to-monocyte ratio (LMR; *p* < 0.001) were significantly lower in the moderate and the severe groups. Among radiographic features, GB wall thickness (*p* < 0.001), HU values of the gallbladder wall (*p* < 0.001), and volume of the GB (*p* < 0.001) were significantly increased in the moderate and severe groups. Additionally, the presence of pericholecystic fluid (*p* < 0.001), gangrenous change (*p* < 0.001), and abscess formation (*p* = 0.007) were more frequently observed in these groups.

### 3.4. Predictors of Complicated Group from Univariate Analysis

We used a univariable logistic regression model to analyze the predictive value of general characteristics, inflammatory markers, and image parameters, as illustrated in Table 3. Age (OR: 1.03; *p* < 0.001), DM (OR = 1.68; *p* = 0.048), hypertension (OR: 1.7; *p* = 0.013), temperature (OR: 2.24; *p* < 0.001), heart rate (OR: 1.04; *p* < 0.001), need for oxygen therapy (OR: 0.185; *p* < 0.001), WBC (OR: 1.15; *p* < 0.001), neutrophil percentage (OR: 0.96; *p* < 0.001), platelet count (OR: 1.00; *p* = 0.002), CRP (OR: 1.19; *p* < 0.001), creatinine level (OR: 5.4; *p* < 0.001), total bilirubin level (OR: 1.56; *p* = 0.017), INR (OR: 6.85; *p* < 0.001), sodium level (OR: 0.812; *p* < 0.001), GB wall thickness (OR: 9.15; *p* < 0.001), HU value of the GB wall (OR: 1.02; *p* < 0.001), volume of the GB (OR: 1.01; *p* < 0.001), the presence of pericholecystic fluid (OR: 5.17; *p* < 0.001), gangrenous change (OR: 4.25; *p* < 0.001), and abscess formation (OR: 6.45; *p* = 0.014) were all candidate predictors for moderate to severe grades of cholecystitis.

### 3.5. Multivariable Models for Identifying the Complicated Group

**Figure 3:**
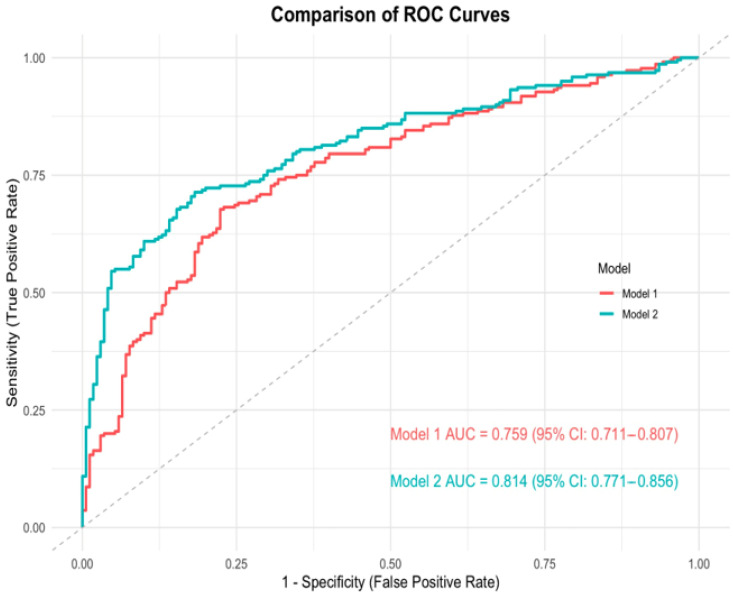
ROC of Model 1 and Model 2.

In the multivariable logistic regression analysis, as presented in Table 4, Model 1, which included only radiographic features, showed that the HU value of the GB wall (OR = 1.01; 95% CI, 1.00–1.02; *p* = 0.018), gangrenous change (OR = 2.10; 95% CI, 1.21–3.71; *p* < 0.01), GB volume (OR = 1.01; 95% CI, 1.00–1.01; *p* < 0.01), and the presence of pericholecystic fluid (OR = 2.34; 95% CI, 1.28–4.36; *p* < 0.01) were significantly associated with complicated acute cholecystitis. Model 1 achieved an area under the curve (AUC) of 0.759 for predicting the complicated group (Figure 3 and Figure S1).

In Model 2, which incorporated both significant imaging and laboratory variables, the GB wall’s HU value (OR = 1.01; 95% CI, 1.00–1.02; *p* = 0.028), gangrenous change (OR = 1.98; 95% CI, 1.08–3.69; *p* = 0.028), elevated WBC count (OR = 1.08; 95% CI, 1.01–1.15; *p* = 0.022), and CRP level (OR = 1.10; 95% CI, 1.06–1.17; *p* < 0.01) remained independent predictors. In contrast, GB wall thickness, volume, and the presence of pericholecystic fluid did not reach statistical significance in this combined model. Model 2 demonstrated improved discriminative ability with an AUC of 0.814 (Figure 3 and Figure S1).

### 3.6. The Novel Scoring System for Predicting ACC

**Figure 4:**
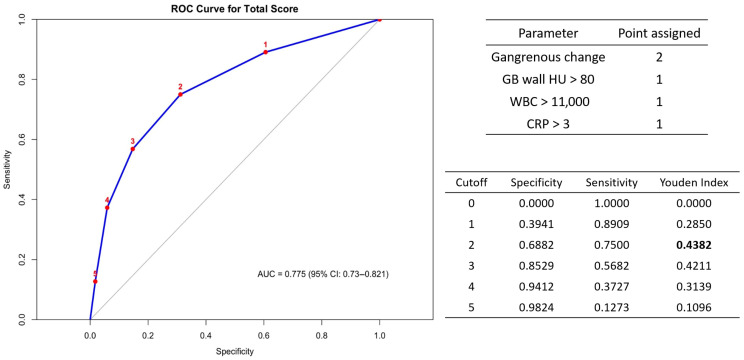
New scoring system represented by Model 3.

Based on the significant predictors in Model 2, we created a novel scoring system by assigning two points for the presence of gangrenous change and one point each for GB wall enhancement > 80 HU, WBC > 11,000 per 10^3^/μL, and CRP > 3 mg/dL, referred to as Model 3. This scoring system demonstrated good diagnostic performance with an AUC of 0.775 (95% CI: 0.73–0.821). The optimal cutoff was identified as a score ≥ 2, yielding the highest Youden index of 0.4382 (43.82%) for differentiating complicated cases of acute cholecystitis (Figure 4).

### 3.7. Decision Tree Analysis for Predicting ACC

**Figure 5:**
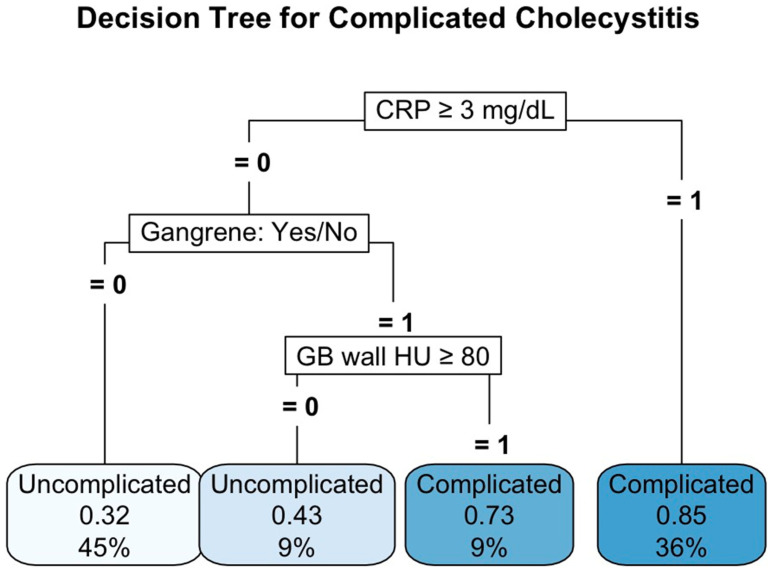
Decision tree analysis of Model 3.

The decision tree analysis identified C-reactive protein (CRP) level, the presence of gangrene, and the gallbladder (GB) wall’s Hounsfield unit (HU) value as key predictors of complicated acute cholecystitis (ACC). Patients with CRP ≥ 3 mg/dL were most likely to have complicated cholecystitis, with a probability of 0.85 (36%). In those with CRP < 3 mg/dL, further classification by gangrene and GB wall HU value refined the prediction: patients without gangrene had a 45% chance of uncomplicated disease (probability 0.32), whereas those with gangrene and GB wall HU value ≥ 80 had a high probability (0.73) of complicated cholecystitis. Overall, the model (Figure 5) provided stratified risk estimates for complication, assisting in early identification and potential management strategies for high-risk patients.

## 4. Discussion

In this study, we aimed to develop an objective and clinically applicable scoring system to facilitate the early prediction of acute complicated cholecystitis (ACC) using contrast-enhanced CT features, laboratory biomarkers, and clinical parameters. Traditionally, treatment strategies for acute cholecystitis in our institution have been based on clinical judgment, guided by the Tokyo Guidelines 2018 (TG18) and supported by history, physical examination, and lab data. However, imaging—particularly CT—has lacked a standardized framework for identifying complicated cases promptly. Our findings demonstrated that specific radiologic features such as GB wall enhancement (Hounsfield units, HU), the presence of gangrenous change, GB volume, and pericholecystic fluid were useful in distinguishing ACC from uncomplicated cases. These observations align with previous studies indicating CT’s utility in evaluating disease severity, particularly when ultrasound may fall short in detecting complications like gangrene or perforation [5,6,7].

While ultrasound remains the first-line imaging modality for gallstone detection and Murphy sign assessment, its diagnostic utility is limited by operator dependency and lower sensitivity for structural complications. In contrast, CT provides a more objective evaluation of GB wall thickening, contrast enhancement, and distention—key features associated with disease severity. Incorporating biomarkers such as WBC count and CRP further enhanced the model’s predictive ability. These markers, widely recognized indicators of inflammation, have been shown to correlate with AC severity and are also emphasized in TG18 [2,3].

Compared with earlier reports, our study contributes more detailed radiologic insights with direct clinical relevance. CT features such as intraluminal gas, GB wall abnormalities, and pericholecystic changes were significantly associated with ACC [2,10], as were mural striation, decreased wall enhancement, and GB distention [10,11,12,13,14]. Though prior studies, such as Chang et al. [12], identified a short-axis GB diameter >4 cm as being predictive of gangrenous cholecystitis, our multivariable analysis did not confirm its significance. Recognizing the gallbladder as a 3D organ, we opted to use GB volume, with >125 mm^3^ emerging as a better predictor. Similarly, we validated a GB wall thickness cutoff of 4 mm, consistent with findings from Kim et al. [13]. While Sureka et al. [14] proposed CT attenuation cutoffs of 31.5 HU for the GB wall and 12.5 HU for bile, our data indicated that a higher threshold of 80 HU was more predictive, potentially reflecting differences in patient selection, as their study included only pathologically confirmed cases and had a small sample size.

Pericholecystic fluid, previously described as a highly specific sonographic marker for ACC (specificity up to 94%) [22], showed significant differences between groups in our univariate analysis. However, it was not retained in the multivariable model. This may be due to its collinearity with other features such as gangrenous change or wall enhancement, or due to variability in measurement sensitivity on CT versus ultrasound.

Although the CT attenuation ratio during the arterial phase (ARAP) has been reported as a strong predictor of gangrenous cholecystitis—with Maehira et al. citing 84.6% sensitivity and 83.9% specificity for ARAP ≥ 1.46 [23]—this variable was not assessed in our study due to protocol limitations. In our emergency department, portal venous phase imaging is standard, and arterial-phase images were often unavailable. Additionally, ARAP calculation requires specialized post-processing and region-of-interest placement, which may be impractical in an emergency setting. Nonetheless, future prospective studies with dynamic CT protocols could further explore ARAP’s clinical utility.

In multivariable analysis, WBC and CRP remained significant predictors, while inflammatory ratios such as the neutrophil-to-lymphocyte ratio (NLR), platelet-to-lymphocyte ratio (PLR), and lymphocyte-to-monocyte ratio (LMR) did not, likely due to multicollinearity. Our WBC cutoff of 11× 10^3^/μL was lower than thresholds proposed by prior studies (9000–18,000/mm^3^) [9,14,24,25,26], and our CRP cutoff of 3 mg/dL was also lower than previous reports (5–29 mg/dL) [24,25,26,27,28,29,30], suggesting that imaging markers may provide greater predictive value than laboratory parameters alone. Similarly, while previous studies have proposed NLR, PLR, and LMR as useful for predicting ACC [24,25,27,31,32,33,34,35], none were independently significant in our model.

Our new scoring system, composed of CT parameters, demonstrated a good area under the curve (AUC) and excellent specificity at a 5-point cutoff, effectively identifying patients with ACC. Both multivariable logistic regression and decision tree analyses consistently highlighted gangrenous change, GB wall enhancement (HU), WBC, and CRP as key predictors. The decision tree further illustrated the hierarchical diagnostic value of these variables and offered a practical, interpretable tool for risk stratification. We recommend the integration of this scoring system into clinical workflows to enhance early, accurate differentiation of complicated versus uncomplicated cholecystitis in emergency care settings.

## 5. Limitations

This study has several limitations. First, as a retrospective study, patients with greater severity were predominantly treated with PTGBD, with delayed cholecystectomy arranged for subsequent hospitalizations, meaning that the final pathologic characteristics may not equally represent the severity of inflammation at the ED presentation. Second, we excluded patients diagnosed with ACC solely by means of ultrasound or non-contrast CT images, despite their proportion being relatively small. As a result, our new scoring system could not be applied to this subgroup. Third, certain laboratory data associated with inflammation, such as monocyte distribution width (MDW) and lactate concentration, were not included in our analysis due to missing data. Additionally, the potential predictive value of procalcitonin for ACC warrants further investigation [36,37,38], despite its less common utilization in ED laboratory testing. Lastly, variations in contrast CT protocols could potentially influence Hounsfield unit (HU) measurements and subsequently affect the results [23].

## 6. Conclusions

This study developed and validated a clinically practical scoring system based on key radiographic and laboratory parameters—gangrenous change, GB wall HU value, WBC, and CRP—for the early identification of complicated acute cholecystitis. The scoring system showed good diagnostic performance with an AUC of 0.775 and demonstrated optimal accuracy at a cutoff score of ≥2. Multivariable logistic regression and decision tree analyses consistently highlighted the same predictors, reinforcing their clinical relevance. By integrating these tools into emergency department workflows, clinicians may improve diagnostic precision, expedite decision-making, and better triage patients at risk for severe disease.

## Figures and Tables

**Table 1:** Demographic characteristics of the study population (N = 390).

Tokyo Grading	Mild	Moderate	Severe	*p* Value
Number, *n* (%)	170	170	50	
Age (years)	56.07 ± 16.06	61.81 ± 16.92	72.38 ± 16.78	<0.001 *
*Sex*				0.026 *
Female, *n* (%)	91 (53.5%)	87 (51.2%)	16 (32%)	
Male, *n* (%)	79 (46.5%)	83 (48.8%)	34 (68%)	
BMI (kg/m^2^)	25.2 ± 3.79	25.36 ± 4.47	24.61 ± 4.54	0.553
*Comorbidities*				
Diabetes mellitus, *n* (%)	27 (15.9%)	32 (18.8%)	22 (44%)	<0.001 *
Hypertension, *n* (%)	54 (31.8%)	68 (40%)	31 (62%)	0.001 *
CKD, *n* (%)	1 (0.6%)	0 (0%)	10 (20%)	<0.001 *
CCI	7.73 ± 75.91	2.49 ± 2.134	24.48 ± 140.79	0.161
*Vital signs at triage*				
Temperature (°C)	36.72 ± 0.63	37.05 ± 0.79	37.41 ± 1.08	<0.001 *
Heart rate (bpm)	79.9 ± 16.48	89.56 ± 18.32	106.5 ± 23.71	<0.001 *
Respiratory rate (bpm)	17.99 ± 7.76	17.46 ± 1.77	18.86 ± 3.05	0.259
MAP (mmHg)	102.60 ± 17.37	99.28 ± 16.66	97.68 ± 20.96	0.104
GCS	15 ± 0	15 ± 0	14.02 ± 1.97	<0.001 *
O_2_ supply, *n* (%)	6 (3.5%)	19 (11.2%)	18 (10.6%)	<0.001 *
FiO_2_ (%)	21.44 ± 2.34	22.2 ± 4.93	31.57 ± 14.58	<0.001 *
Inotrope use, *n* (%)	0 (0%)	0 (0%)	12 (24%)	<0.001 *
*Outcomes*				
Length of stay (days)	5.59 ± 2.86	7.02 ± 4.22	12.54 ± 10.16	<0.001 *
ERCP, *n* (%)	27 (15.9%)	34 (20%)	11 (22%)	0.52
PTGBD, *n* (%)	18 (10.6%)	55 (32.4%)	28 (16.5%)	<0.001 *
Surgery, *n* (%)	94 (55.3%)	72 (42.4%)	6 (12%)	<0.001 *
ICU admission, *n* (%)	5 (2.9%)	20 (11.8%)	18 (36%)	<0.001 *
Mortality, *n* (%)	0 (0%)	0 (0%)	3 (6%)	0.002 *

Abbreviations: n, number; BMI, body mass index; CKD, chronic kidney disease; CCI, Charlson comorbidity index; MAP, mean arterial pressure; GCS, Glasgow coma scale; ERCP, endoscopic retrograde cholangiopancreatography; PTGBD, percutaneous transhepatic gallbladder drainage; ICU, intensive care unit. * *p* < 0.05.

**Table 2:** Lab data and image characteristics of the study population (N = 390).

Tokyo Grading	Mild (*n* = 170)	Moderate (*n* = 170)	Severe (*n* = 50)	*p* Value
*Lab data*				
WBC (per 10^3^/μL)	9.79 ± 3.21	13.10 ± 5.27	12.40 ± 6.00	<0.001 *
Platelet (per 10^3^/μL)	236.49 ± 76.65	229.36 ± 75.56	186.22 ± 92.60	<0.001 *
Neutrophil (%)	71.24 ± 14.08	79.19 ± 10.99	81.25 ± 12.00	<0.001 *
CRP (mg/dL)	1.59 ± 3.82	8.52 ± 10.73	13.95 ± 13.33	<0.001 *
Creatinine (mg/dL)	0.82 ± 0.24	0.89 ± 0.32	2.7 ± 3.39	<0.001 *
Bilirubin T^+^ (mg/dL)	0.94 ± 1.09	1.53 ± 1.69	1.93 ± 1.30	<0.001 *
Lactate (mg/dL)	17.07 ± 10.82	16.81 ± 7.93	27.03 ± 21.46	0.03 *
Na (mEq/L)	138.61 ± 2.47	136.8 ± 3.72	135.8 ± 3.35	<0.001 *
K (mEq/L)	3.79 ± 0.37	3.69 ± 0.37	3.9 ± 0.055	<0.001 *
INR	1.02 ± 0.10	1.08 ± 0.12	1.39 ± 0.51	<0.001 *
NLR	6.63 ± 6.94	12.63 ± 15.42	20.6 ± 22.05	<0.001 *
PLR	19.53 ± 20.91	32.24 ± 38.89	37.79 ± 35.56	<0.001 *
LMR	2.94 ± 1.58	1.87 ± 1.27	1.94 ± 2.71	<0.001 *
*Image parameter*				
GB wall thickness (mm)	4.22 ± 2.50	6.74 ± 4.32	5.85 ± 2.92	<0.001 *
GB wall HU	60.51 ± 26.53	74.64 ± 26.36	73.5 ± 26.65	<0.001 *
GB volume (mm^3^)	99.72 ± 51.84	122.68 ± 66.04	123.76 ± 72.73	<0.001 *
Gallstone present, n (%)	98 (57.6%)	97 (57.1%)	21 (42%)	0.124
Multiple Gallstones, n (%)	59 (34.7%)	53 (31.2%)	15 (30%)	0.736
Sludge present, n (%)	18 (10.6%)	26 (15.3%)	7 (14%)	0.43
Gallstone over GB outlet, *n* (%)	58 (34.1%)	64 (37.6%)	12 (24%)	0.202
Pericholecystic fluid, *n* (%)	106 (62.4%)	157 (92.4%)	40 (80%)	<0.001 *
Abscess formation, *n* (%)	2 (1.2%)	12 (7.1%)	4 (8%)	0.007 *
Gangrene change, *n* (%)	38 (22.4%)	99 (58.2%)	24 (48%)	<0.001 *
GB perforation, *n* (%)	2 (1.18%)	13 (7.64%)	4 (8%)	0.0053

Abbreviations: n, number; WBC, white blood cell; CRP, C-reactive protein; Bilirubin T, total bilirubin; INR, international normalized ratio; NLR, neutrophil-lymphocyte ratio; PLR, platelet-lymphocyte ratio; LMR, lymphocyte-monocyte ratio; GB, gallbladder; HU, Hounsfield unit; CBD, common bile duct * *p* < 0.05.

**Table 3:** Predictors of complicated acute cholecystitis for 390 patients: univariate analysis.

Characteristics	Odds Ratio (95% CI)	*p* Value
Age	1.03 (1.02–1.04)	<0.0001 *
Sex	0.76 (0.51–1.14)	0.189
Diabetes mellitus	1.68 (1.01–2.81)	0.048 *
Hypertension	1.70 (1.12–2.58)	0.013 *
CKD	7.88 (0.99–62.15)	0.05
O_2_ supply	0.185 (0.08–0.45)	<0.0001 *
*Vital signs*		
Temperature (°C)	2.24 (1.61–3.12)	<0.0001 *
Heart rate (bpm)	1.04 (1.03–1.06)	<0.0001 *
FiO_2_ (%)	1.12 (1.05–1.2)	0.001 *
GCS	0	0.096
*Lab data*		
WBC (per 10^3^/μL)	1.15 (1.1–1.21)	<0.0001 *
Platelet (per 10^3^/μL)	1.00 (0.99–1.00)	0.002
Neutrophil (%)	0.96 (0.93–1.00)	<0.0001 *
CRP (mg/dL)	1.19 (1.13–1.25)	<0.0001 *
Creatinine (mg/dL)	5.4 (2.48–11.78)	<0.0001 *
Bilirubin T (mg/dL)	1.56 (1.27–1.98)	<0.0001 *
INR	6.85 (0.48–97.74)	<0.0001 *
Na (mEq/L)	0.812 (0.75–0.88)	<0.0001 *
K (mEq/L)	0.75 (0.45–1.24)	0.262
*Image parameter*		
GB wall thickness (mm)	9.15 (4.14–20.2)	<0.0001 *
GB wall HU	1.02 (1.01–1.03)	<0.0001 *
GB volume (mm^3^)	1.01 (0.99–1.01)	<0.0001 *
Pericholecystic fluid, *n* (%)	5.17 (3.08–8.95)	<0.0001 *
Gangrene change, *n* (%)	4.25 (0.72–6.25)	<0.0001 *
Abscess formation, *n* (%)	6.45 (1.46–28.44)	0.014 *

Abbreviations: CKD, chronic kidney disease; GCS, Glasgow coma scale; WBC, white blood cell; CRP, C-reactive protein; Bilirubin T, total bilirubin; INR, international normalized ratio; GB, gallbladder; HU, Hounsfield unit. * *p* < 0.05.

**Table 4:** Multivariate analysis in the complicated group.

Characteristics	Original Model 1 OR (95% CI)	*p* Value	Original Model 2 OR (95% CI)	*p* Value
GB wall thickness (mm)	1.90 (0.791–4.92)	0.166	1.25 (0.48–3.42)	0.649
GB wall HU	1.01 (1.00–1.02)	<0.018 *	1.01 (1.00–1.02)	0.028 *
Gangrene change, *n* (%)	2.10 (1.21–3.71)	<0.01 *	1.98 (1.08–3.69)	0.028 *
GB volume (mm^3^)	1.01 (1.00–1.01)	<0.01 *	1.00 (0.99–1.01)	0.355
Pericholecystic fluid	2.34 (1.28–4.36)	<0.01 *	1.68 (0.892–3.20)	0.112
WBC (per 10^3^/μL)			1.08 (1.01–1.15)	0.022 *
CRP (mg/dL)			1.10 (1.06–1.17)	<0.01 *

Abbreviations: GB, gallbladder; HU, Hounsfield unit; WBC, white blood cell; CRP, C-reactive protein; Bilirubin T, total bilirubin * *p* < 0.05.

## Data Availability

The data that support the findings of this study are not publicly available due to restrictions imposed by the Taiwan Personal Data Protection Act. Researchers who wish to access the raw data may submit a request to the Office of Human Research and the Institutional Review Board (IRB) of Taipei Medical University Hospital (e-mail: ohr@tmu.edu.tw).

## Supplementary Materials

The following supporting information can be downloaded at: https://www.mdpi.com/article/10.3390/diagnostics15141777/s1, Figure S1: ROC of variables in Model 1 and 2; Table S1: ICD10; Table S2: Severity grading of TG18.

## List of Abbreviations

GB	Gallbladder
ED	Emergency department
ACC	Acute complicated cholecystitis
LOS	Length of stay
CT	Computed tomography
MDCT	Multidetector computed tomography
PTGBD	Percutaneous transhepatic gallbladder drainage
TG18	Tokyo Guideline 2018
ICD–10–CM	International Statistical Classification of Diseases, Tenth Revision, Clinical Modification
GCS	Glasgow coma scale score
CCI	Charlson comorbidity index
HU	Hounsfield units
ANOVA	Analysis of Variance
ORs	Odds ratios
CI	Confidence intervals
AUC	Area under the curve
ROC	Receiver operating characteristic
CART	Classification and regression tree
DM	Diabetes mellitus
CKD	Chronic kidney disease
FiO2	Mean fraction of inspired oxygen
WBC	White blood cell
CRP	C-reactive protein
NLR	Neutrophil-to-lymphocyte ratio
LMR	Lymphocyte-to-monocyte ratio
