# Supplementary material for: Clinical and Imaging Characteristics to Discriminate Between Complicated and Uncomplicated Acute Cholecystitis: A Regression Model and Decision Tree Analysis
Source: Diagnostics (Basel). 2025 Jul 14;15(14):1777. doi: 10.3390/diagnostics15141777 (PMC12293178; doi:10.3390/diagnostics15141777)
Supplement: Supplementary file 1 [file diagnostics-15-01777-s001.zip › Table S2. Severity grading of TG18.pdf]

Table S2

**TG18 severity grading for acute cholecystitis**
Grade III (severe) acute cholecystitis<br>Grade III acute cholecystitis is associated with dysfunction of any one of the following organs/systems:<br>1. Cardiovascular dysfunction: hypotension requiring treatment with Dopamine ≥ 5μg/kg per min, or any dose of norepinephrine<br>2. Neurological dysfunction: decreased level of consciousness<br>3. Respiratory dysfunction: PaO_2/FiO_2 ratio < 300<br>4. Renal dysfunction: oliguria, creatinine > 2.0 mg/dl<br>5. Hepatic dysfunction: PT-INR > 1.5<br>6. Hematological dysfunction: platelet count < $100,000/mm^3$
Grade II (moderate) acute cholecystitis<br>Grade II acute cholecystitis is associated with any one of the following conditions:<br>1. Elevated WBC count (> 18,000/mm3)<br>2. Palpable tender mass in the right upper abdominal quadrant<br>3. Duration of complaints > 72 hours<br>4. Marked local inflammation (gangrenous cholecystitis, pericholecystic. abscess, hepatic abscess, biliary peritonitis, emphysematous cholecystitis)
Grade I (mild) acute cholecystitis<br>Grade I acute cholecystitis does not meet the criteria of Grade III or Grade II acute cholecystitis. It can also be defined as acute cholecystitis in a healthy patient with no organ dysfunction and mild inflammatory changes in the gallbladder, making cholecystectomy a safe and low-risk operative procedure

Abbreviations: TG18, Tokyo Guideline 2018; PaO_2, partial pressure of oxygen in alveoli; FiO_2, fracture of inspired oxygen; PT-INR, prothrombin time- international normalized ratio; WBC, white blood cell
