# Supplementary figures and images for: Clinical and Imaging Characteristics to Discriminate Between Complicated and Uncomplicated Acute Cholecystitis: A Regression Model and Decision Tree Analysis
Source: Diagnostics (Basel). 2025 Jul 14;15(14):1777. doi: 10.3390/diagnostics15141777 (PMC12293178; doi:10.3390/diagnostics15141777)

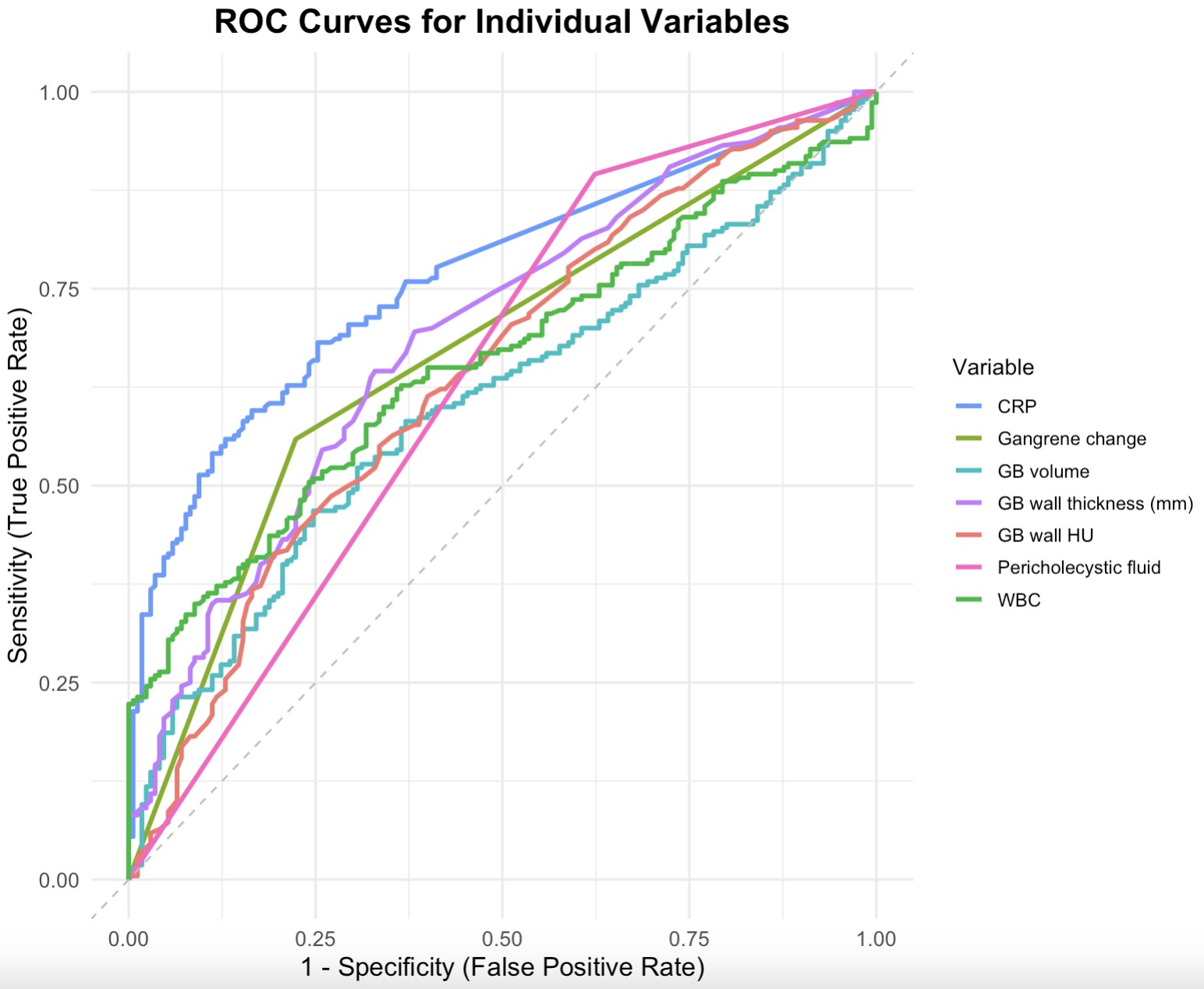

Supplement: Supplementary file 1 [file diagnostics-15-01777-s001.zip › Figure S1. ROC of variables in Model 1 and 2.tif]
